# Shen Shuai II Recipe Attenuates Renal Interstitial Fibrosis by Improving Hypoxia via the IL-1*β*/*c-Myc* Pathway

**DOI:** 10.1155/2021/5539584

**Published:** 2021-06-07

**Authors:** Liuyi Yang, Meng Wang, Yuan Zhou, Jing Yang, Chaoyang Ye, Chen Wang

**Affiliations:** ^1^Department of Nephrology, Shuguang Hospital Affiliated to Shanghai University of Traditional Chinese Medicine, Shanghai 201203, China; ^2^Key Laboratory of Liver and Kidney Diseases, Ministry of Education, Shanghai University of Traditional Chinese Medicine, Shanghai 201203, China; ^3^TCM Institute of Kidney Disease, Shanghai University of Traditional Chinese Medicine, Shanghai 201203, China; ^4^Shanghai Key Laboratory of Traditional Chinese Clinical Medicine, Shanghai University of Traditional Chinese Medicine, Shanghai 201203, China

## Abstract

**Background:**

Renal interstitial fibrosis is a pathological manifestation of progression of chronic kidney disease induced by various factors. Shen Shuai II Recipe (SSR) has been used in clinical practice for more than 20 years, and clinical studies have confirmed that SSR significantly improves the renal function of patients with chronic kidney disease. However, the specific mechanisms underlying its efficacy require further research. This study aims to explore the influencing factors of renal interstitial fibrosis in the context of hypoxia via the IL-1*β*/*c-Myc* pathway and the potential molecular mechanisms of SSR intervention in vivo and in vitro.

**Methods:**

A rat model of chronic renal failure was developed by performing 5/6 (ablation/infarction, A/I) surgery on randomly selected, male Sprague Dawley rats. Thirty-six successfully modeled rats were randomly divided into three groups: 5/6 (A/I), 5/6 (A/I) + SSR, and 5/6 (A/I) + losartan. Another 12 rats were used as the sham group. After 8 weeks of the corresponding intervention, renal function, liver function, and protein expression of renal-fibrosis-related factors, HIF-1*α*, IL-1*β*, and *c-Myc*, were detected. In vitro analysis was performed using hypoxia-induced rat renal tubular epithelial cells (NRK-52E) and IL-1*β*-stimulated rat renal interstitial fibroblasts (NRK-49F). IL-1*β* concentration in the culture medium and IL-1*β* protein expression in hypoxic NRK-52E treated with different concentrations of SSR were investigated. Furthermore, we also studied the changes in protein expression of *c-Myc* and fibrosis-related factors after *c-Myc* gene silencing in IL-1*β*-stimulated NRK-49F treated with SSR.

**Results:**

Shen Shuai II Recipe significantly reduced RIF and downregulated the expression of HIF-1*α*, *c-Myc*, and IL-1*β* proteins in 5/6 (A/I) rats with chronic renal failure. It also inhibited IL-1*β* secretion from NRK-52E induced by hypoxia, which in turn inhibited fibroblast activation mediated by the IL-1*β*/*c-Myc* pathway, and finally reduced the overproduction of the extracellular matrix.

**Conclusion:**

The renoprotective effects of SSR in rats with chronic renal failure may be related to its inhibition of hypoxia via the IL-1*β*/*c-Myc* pathway. Thus, SSR is a potentially effective drug for delaying the progression of renal interstitial fibrosis.

## 1. Introduction

Chronic kidney disease (CKD), a major global health problem with high morbidity and mortality, places a huge economic burden on families and society [1, 2]. Renal interstitial fibrosis (RIF) caused by activation of fibroblasts and imbalance of synthesis and degradation of the extracellular matrix (ECM) is the main reason for the continuous progression of various types of CKD [3].

Oxygen plays an important role in the metabolism, energy production, and equilibrium of the internal environment of the body. The physiological characteristics of high oxygen consumption and high metabolism make the kidney vulnerable to a hypoxic microenvironment [4]. Long-term hypoxia leads to RIF, and RIF further inhibits the diffusion and supply of oxygen to renal tubules and interstitial cells, which causes apoptosis or epithelial-mesenchymal transdifferentiation, which in turn aggravates renal fibrosis and subsequent chronic hypoxia, thus forming a vicious circle with end-stage renal disease as the end point; therefore, chronic hypoxia is a final common pathway to end-stage renal failure [5]. The oxygen consumption of renal tubular cells accounts for more than 80% of the total oxygen consumption of the kidney; therefore, renal tubular cells are particularly sensitive to hypoxia. Hypoxia-inducible factor-1*α* (HIF-1*α*) is mostly expressed in renal tubular cells, and HIF-1*α* is also a key transcription factor that regulates various cellular hypoxia responses, including inflammatory responses [6, 7].

Excessive inflammation is also an initiating factor of fibrotic diseases [8]. Among proinflammatory cytokines, interleukin-1*β* (IL-1*β*) is the central link between acute and chronic inflammatory response and fibrosis. In RIF, the oncogene protein *c-Myc* is highly expressed in renal fibroblasts and is closely associated with the proliferation and activation of fibroblasts [9]. A previous study has found that a *c-Myc* inhibitor blocked IL-1*β*-induced fibroblast activation [10]. A recent study has confirmed that the IL-1*β*/*c-Myc* pathway plays a vital role in renal fibrosis by regulating cell proliferation and differentiation [11].

Several studies have confirmed that traditional Chinese medicine (TCM), which has a long history, is safe and reliable for long-term treatment of patients with CKD [12, 13]. According to TCM, the basic pathological feature of CKD is spleen-kidney deficiency, and qi stagnation and blood stasis is a key factor in the progression of CKD [14]. On the basis of this concept, Professor Wang Chen established—more than 20 years ago—the TCM prescription Shen Shuai II Recipe (SSR) to invigorate the spleen and kidney, activate blood circulation, and remove blood stasis. Shen Shuai II Recipe is an effective clinical prescription for the treatment of patients with CKD. Previous clinical studies have confirmed that SSR effectively improves renal function, renal anemia, and microinflammation in patients with CKD stage 3-4 [15–17]. Experimental studies have also confirmed that SSR plays a certain role in improving renal fibrosis by increasing renal blood flow and improving intrarenal hypoxia in 5/6 renal ablation/infarction (A/I) rats [18, 19]. However, there is no research on the specific mechanism of SSR in renal hypoxia, inflammation, and RIF. Therefore, the present study aimed to investigate whether SSR alleviates RIF by regulating hypoxia via the IL-1*β*/*c-Myc* pathway in vivo and in vitro.

## 2. Materials and Methods

### 2.1. Drugs

The composition of SSR is shown in Table 1. The raw herbs were purchased from Shanghai Kangqiao Chinese Medicine Tablet Co., Ltd. and identified by Dr. Guanglin Xu from the Department of Pharmacy, Shuguang Hospital affiliated to Shanghai University of Traditional Chinese Medicine (SHUTCM). The SSR was prepared as previously described [19], and the final concentration of the original drug was 4 g/mL. The positive control group losartan (100 mg per tablet) was purchased from Hangzhou Mosadong Pharmaceutical Co., Ltd. (Batch number: N035513) and dissolved in deionized water to make a suspension with a concentration of 3.33 mg/mL. The medicinal mixture was extracted twice with 75% ethanol and filtered, and the ethanol extract was evaporated and dehydrated in vacuum. The extract powder (0.44 kg) was dissolved in Dulbecco's modified Eagle's medium (DMEM) at the desired concentration for in vitro experiment.

### 2.2. 5/6 (A/I) Rat Model and Animal Study Protocol

Eight-week-old, specific-pathogen-free, healthy, male Sprague Dawley (SD) rats weighing 140 g to 160 g were purchased from Shanghai Sipur-Bikai Experimental Animal Co., Ltd. and were raised in the Experimental Animal Center of SHUTCM. The rearing temperature was 20–24°C (relative humidity 53–57% and a light-dark cycle of 12/12 h). After a week of adaptive feeding, a rat model of chronic renal failure with 5/6 (A/I) was created as previously described [19, 20]. Four weeks after the operation, 36 successful model rats were randomly divided into three groups: a 5/6 (A/I) group (saline 3 mL/day by gavage), 5/6 (A/I) + SSR group (SSR 3 mL/day by gavage), and 5/6 (A/I) + losartan group (losartan potassium 3 mL/day by gavage); in addition, 12 SD rats were used as the sham operation group (saline 3 mL/day by gavage). All rats were euthanized with sodium pentobarbital (40 mg/kg; intraperitoneal) after 8 weeks of intervention. This experimental procedure was approved by the Ethics Committee of SHUTCM in accordance with the principles outlined in the NIH Guide for the Care and Use of Laboratory Animals.

### 2.3. Chemical Analysis of Shen Shuai II Recipe Extract by Ultra-High-Performance Liquid Chromatography-High-Resolution Mass Spectrometry

The chemical components of SSR were analyzed and determined using a Dionex UltiMate 3000-Q Exactive ultra-high-performance liquid chromatography-high-resolution mass spectrometer. The optimization parameters of mass spectrometry were as follows: the spray voltage was set at 3.2 kV for the positive ion mode and 2.8 kV for the negative ion mode; sheath gas voltage was 35 arb units; auxiliary gas pressure was 10 arb units; capillary temperature was 320°C; ion source heating temperature was 300°C; and mass range was 80–1200 m/z. Chromatographic separation was achieved at 45°C on a Thermo Scientific Syncronis C18 (150 × 2.1 mm; 3 *µ*m) column equipped with gradient elution. The flow rate was 0.3 mL/min, and the injection volume was 3 *µ*L; the mobile phase consisted of 0.1% formic acid water (C)-acetonitrile (D). The gradient was programmed as follows: 0–12 min, 95% C; 12–14 min, 5% C; and 14.1–16 min, 95% C.

### 2.4. Serum Biochemical Detection

Renal function and liver function were measured before and at the end of the 8-week treatment. Blood samples were obtained from the medial canthus and abdominal aorta of rats, and the serum was collected by centrifuging the blood samples at 3000 rpm for 10 min. The concentration of serum creatinine (Scr), UREA, alanine aminotransferase (ALT), and aspartate transaminase (AST) were determined using an automatic analyzer (AU5800, Beckman, USA).

### 2.5. Western Blotting

Immunoblotting was performed as previously described [19]. In this study, the primary antibodies used were antifibronectin (anti-FN; 1 : 1000; ABCAM, UK), anticollagen-I (anti-COL-I; 1 : 1000, ABCAM; UK), antiproliferating cell nuclear antigen (anti-PCNA; 1 : 1000; Abclonal, China), anti-HIF-1*α* (1 : 1000; Abclonal, China), anti-IL-1*β* (1 : 1000; Abclonal, China), anti-*c-Myc* (1 : 1000; ABCAM, UK), antifibronectin (anti-FN; 1 : 1000; ABCAM, UK), and anticollagen-III (anti-COL-III; 1 : 1000; BOSTER, China). GAPDH (1 : 2000; Proteintech, USA) was used as the loading control.

### 2.6. Histopathological Examination

After soaking the renal tissue in formalin for 24 h, 3 *µ*m sections of paraffin-embedded tissue were stained with hematoxylin and eosin (HE) according to the standard protocol. The histopathological changes were observed under the microscope (Nikon Eclipse 80i, Japan) at 200x magnification.

### 2.7. Enzyme-Linked Immunosorbent Assay

After 8 weeks of treatment, IL-1*β* concentration in rat serum was detected by ELISA (EIA-3418, Elisa Biotech, China). The levels of NRK-52E cell supernatant samples were determined using the Rat IL-1*β* ELISA Kit (BPE30419, Langton Biotech, China) according to the manufacturer's guidelines.

### 2.8. Cell Culture and Treatment

NRK-52E and NRK-49F cells (obtained from the National Infrastructure of Cell Line Resource, Chinese Academy of Medical Sciences) were cultured in DMEM (C11995500, Gibco, China) with 5% or 10% fetal bovine serum (10099–141, Thermo Fisher, USA) and 1% penicillin-streptomycin and incubated at 37°C and 5% CO_2_. When the cell density reached 50%, the cells were cultured in a serum-free medium for 12 h and then treated with different concentrations of SSR according to the results of the cell counting kit-8 (CCK8) assay: NRK-52E: SSL (200 mg/L), SSM (400 mg/L), and SSH (800 mg/L) and NRK-49F: SSL (200 mg/L) and SSH (400 mg/L). Simultaneously, other stimuli were used in this study: NRK-52E cells were subjected to hypoxia (1% oxygen, 94% nitrogen, and 5% carbon dioxide), whereas NRK-49F cells were treated with IL-1*β* (10 ng/mL; PeproTech EC Ltd., UK).

### 2.9. Cell Counting Kit-8 Assay

NRK-52E and NRK-49F cells in the logarithmic growth phase were digested with 0.25% trypsin and seeded into 96-well plates (5 × 10^3^ cells/well) overnight in an incubator at 37°C and 5% CO_2_. After being cultured in a serum-free culture medium for 12 h, SSR at different concentrations was added; after 24 h, the culture medium was replaced with DMEM containing 10% CCK8 solution. After 2 h of incubation, the absorbance was measured at 450 nm using a cell imaging multifunction detection system (Cytation3, BioTek, USA), and the cell viability rate was calculated according to the manufacturer's instructions.

### 2.10. *c-Myc* siRNA Transfection

NRK-49F cells were transfected with a small interference RNA (siRNA) targeting *c-Myc* (20 *µ*m; Shanghai GenePharma Co., Ltd, China).

The sense sequence for rat *c-Myc* siRNA is 5ʹ-GGAAACGGCGAGAACAGUUTT-3ʹ, and the antisense sequence is 5ʹ-AACUGUUCUCGCCGUUUCCTT-3ʹ. The transfection reagent Lipofectamine 3000 was used according to the manufacturer's instructions. The cells were incubated with Lipofectamine 3000 and the target RNA in the Opti-MEM reduced serum medium (31985062, Thermo Fisher, USA) for 12 h. Then, the medium was replaced with a serum-free culture medium for 12 h, and the *c-Myc* gene-silenced NRK-49F cells were cocultured with IL-1*β* and SSR for another 24 h.

### 2.11. Statistical Analysis

SPSS 21.0 software was used to analyze the experimental data; all data are expressed as mean ± standard error of mean (SEM). One-way analysis of variance (ANOVA) and least significant difference multiple comparisons were used to analyze the data between groups. *P* < 0.05 indicated a statistically significant difference between the experimental data.

## 3. Results

### 3.1. Ultra-High-Performance Liquid Chromatography-Mass Spectrometry of Shen Shuai II Recipe

**Figure 1:**
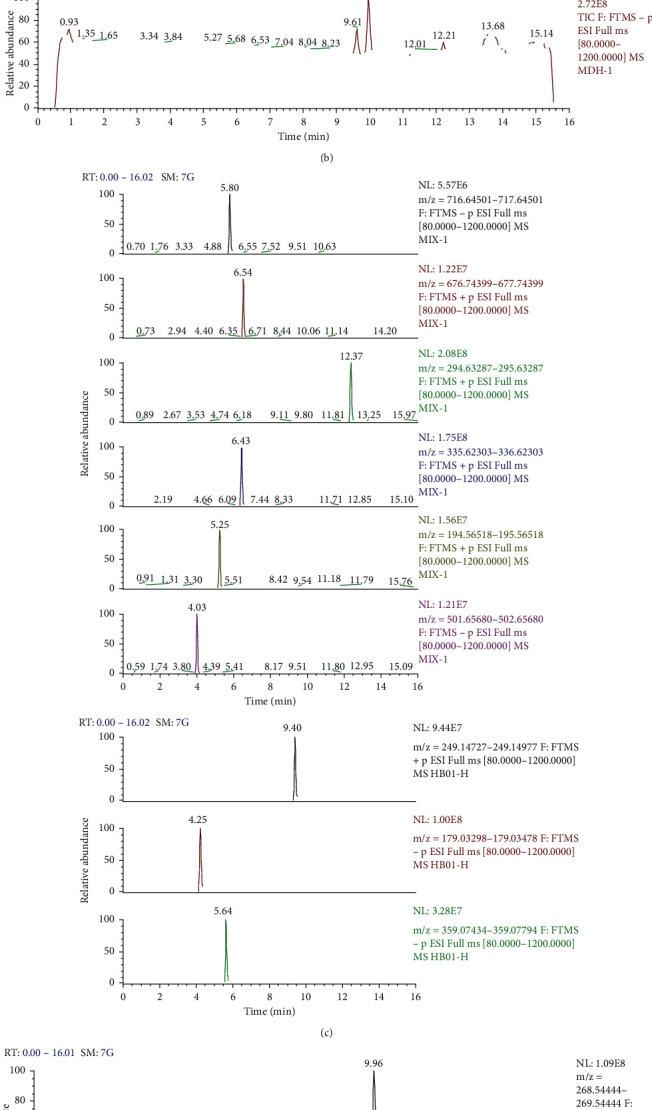
High-performance liquid chromatography-mass spectrometry of Shen Shuai II Recipe. (a) Total ion flow diagram of emodin. (b) Mixed with reference substance of emodin. (c) Flow diagram of positive and negative ions of the mixed control substance. The sequence is as follows: salvianolic acid B icariin, tanshinone IIA, berberine hydrochloride, ferulic acid, amygdalin, codonopsis lactone, caffeic acid, and rosmarinic acid. (d) Anion flow diagram of emodin. (e) Positive and negative ion flow diagrams: codonopsis lactone, caffeic acid, and rosmarinic acid.

Salvianolic acid B, berberine hydrochloride, ferulic acid, icariin, tanshinone IIA, amygdalin, emodin, Codonopsis lactone, caffeic acid, and rosmarinic acid in RSS were separated and quantified. The ions of target compounds icariin ([M + H]+: m/z 677.24399; characteristic component of *Epimedium*), tanshinone IIA([M + H]+: m/z 295.13287; characteristic component of *Salvia miltiorrhiza*), berberine hydrochloride ([M + H]+: m/z 336.12303; characteristic component of *Coptis chinensis*), ferulic acid ([M + H]+: m/z 195.06518; characteristic component of *Ligusticum chuanxiong* and *Angelica sinensis*), amygdalin ([M + H]+: m/z 458.16568; characteristic component of peach kernel), salvianolic acid B ([M + H]+: m/z 719.16066; characteristic component of *Salvia miltiorrhiza*), emodin ([M + H]+: m/z 271.06009; characteristic component of Rheum), Codonopsis lactone ([M + H]+: m/z 249.14852; characteristic component of *Codonopsis pilosula*), caffeic acid ([M + H]+: m/z 181.04953; characteristic component of *Folium perillae*), and rosmarinic acid ([M + H]+: m/z 361.09179; characteristic component of *Folium perillae*) were extracted for quantitative and qualitative analysis. The concentration of salvianolic acid B, berberine hydrochloride, ferulic acid, icariin, and tanshinone IIA, amygdalin, emodin, Codonopsis lactone, caffeic acid, and rosmarinic acid in SSR extract were determined as 1852.35, 503.07, 127.50, 1055.30, 4.92, 6588.04, 110.76, 130.00, 1390.00, and 21280.00 *µ*g/g, respectively (Table 2; Figure 1).

### 3.2. Shen Shuai II Recipe Attenuated Renal Injury and Fibrosis in 5/6 (A/I) Rats without Hepatotoxicity

**Figure 2:**
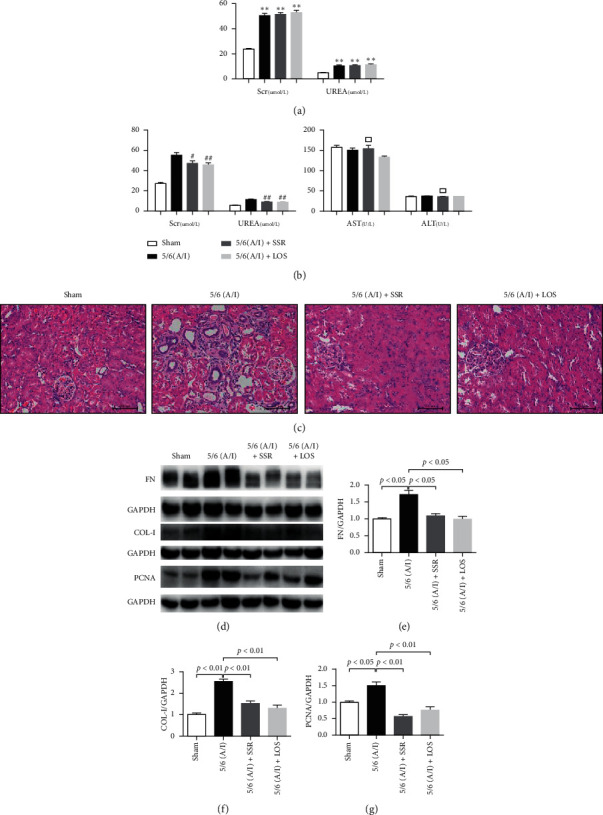
Shen Shuai II Recipe reduces renal fibrosis in 5/6 ablation/infarction (A/I) rats without hepatotoxicity. (a) Concentration of serum creatinine (Scr) and BUN before treatment (*n* = 6). (b) Concentration of Scr, BUN, aspartate transaminase (AST), and alanine aminotransferase (ALT) after treatment (*n* = 6). (c) Representative photomicrographs of HE. Original magnification, ×200. (d) Representative western blotting bands of fibronectin (FN), collagen-I (COL-I), and proliferating cell nuclear antigen (PCNA) proteins in the renal tissue. (e–g) Target protein/GAPDH ratios, including FN/GAPDH, COL-I/GAPDH, and PCNA/GAPDH ratios (*n* = 4). Values are mean ± SE. ^*∗*^*P* < 0.05, ^*∗∗*^*P* < 0.01 vs. the sham group; ^#^*P* < 0.05, ^##^*P* < 0.01 vs. the 5/6 (A/I) group; and ^□^*P* > 0.05 vs. the sham group.

Four weeks after the establishment of the rat model, Scr and UREA of the operated rats significantly increased (Figure 2(a)), indicating that the model was successful. The blood biochemical detection results after treatment suggested that renal indexes such as Scr and UREA were significantly higher in the model group than in the other groups. There was no significant difference in the levels of ALT and AST among different groups (Figure 2(b)). Kidney pathology clearly showed renal fibrosis, with significant inflammatory cell infiltration in the model group (Figure 2(c)). In contrast, renal function was significantly improved in the SSR group; pathological staining showed a lower degree of inflammatory cell infiltration in the SSR group than in the model group. Immunoblotting results showed that the expression of FN and COL-I proteins, two markers of RIF, and PCNA was significantly upregulated in the model group (Figures 2(d)–2(g)). Conversely, SSR treatment markedly decreased the expression of FN, COL-I, and PCNA proteins in 5/6 (A/I) rats (Figures 2(d)–2(g)). Collectively, these data illustrate that SSR safely improves renal interstitial fibrosis in the 5/6 (A/I) rats.

### 3.3. Shen Shuai II Recipe Inhibited the IL-1*β*/*c-Myc* Pathway in 5/6 (A/I) Rats

**Figure 3:**
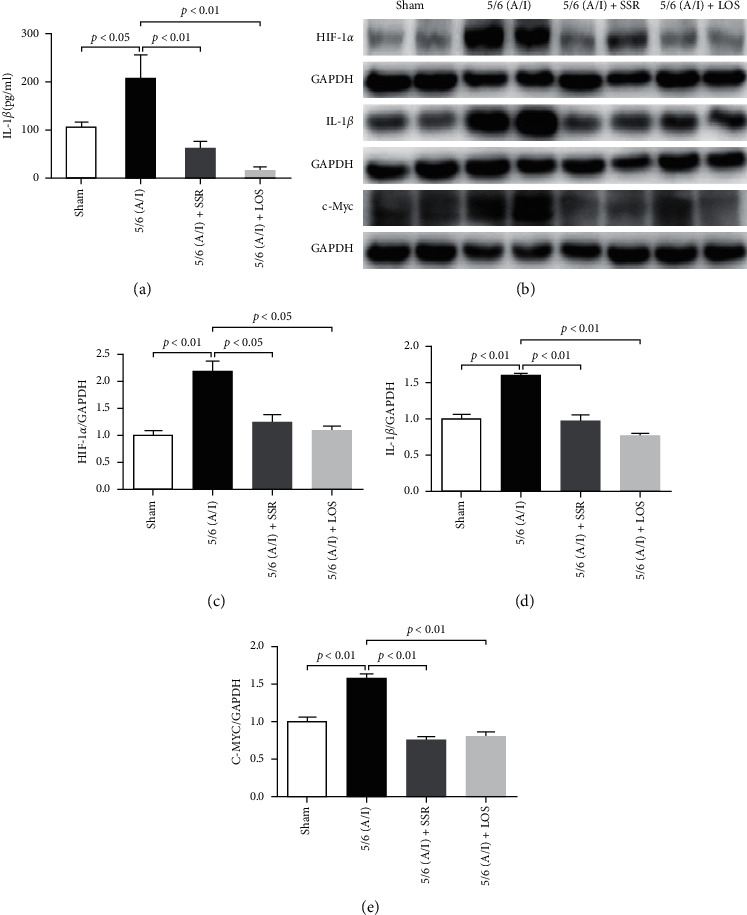
Shen Shuai II Recipe (SSR) ameliorates hypoxia via the interleukin- (IL-) 1*β*/*c-Myc* pathway in 5/6 ablation/infarction (A/I) rats. (a) Concentration of IL-1*β* (*n* = 8). (b). Representative western blotting bands of hypoxia-inducible factor-1*α* (HIF-1*α*), IL-1*β*, and *c-Myc* protein in the renal tissue. (c–e) Target protein/GAPDH ratios, including HIF-1*α*/GAPDH, IL-1*β*/GAPDH, and *c-Myc*/GAPDH ratios (*n* = 4). Values are mean ± SE.

The concentration of IL-1*β* in rat serum was detected by ELISA; it was found that the concentration of IL-1*β* in the model group was significantly higher than that in the SSR group (Figure 3(a)). Western blotting showed that the protein expression of HIF-1*α*, IL-1*β*, and *c-Myc* was significantly higher in the model group than in the SSR group (Figures 3(b), 3(c)). The abovementioned results suggest that SSR may improve hypoxia by inhibiting the IL-1*β*/*c-Myc* pathway.

### 3.4. Shen Shuai II Recipe Inhibited IL-1*β* Expression in NRK-52E Cells under Hypoxia

**Figure 4:**
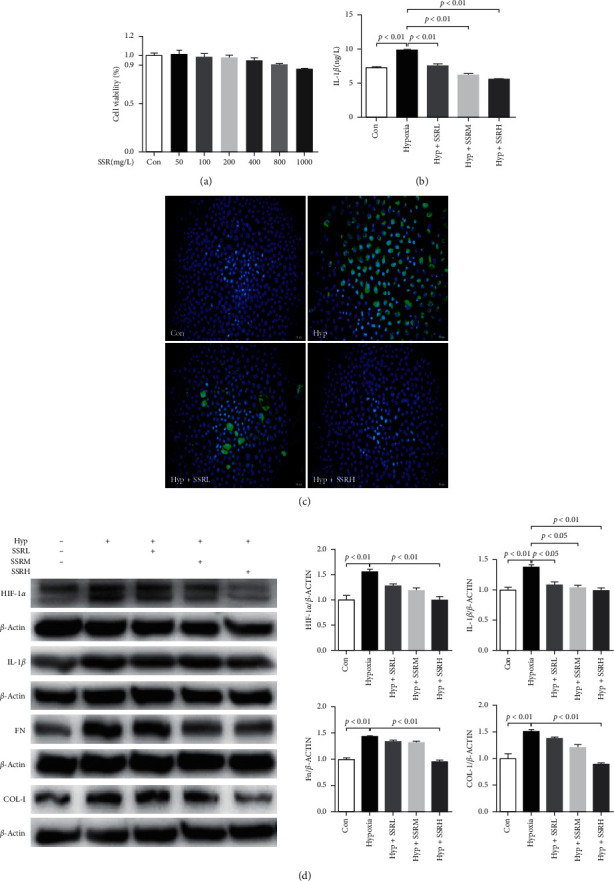
Shen Shuai II Recipe (SSR) inhibits interleukin- (IL-) 1*β* expression in NRK-52E cells under hypoxia. (a) Effect of SSR on the viability of NRK-52E cells (*n* = 5). (b) Concentration of IL-1*β* (*n* = 4). (c) Representative photomicrographs of IL-1*β* expression detected by immunocytochemistry. Original magnification, ×200. (d) Representative western blotting bands of hypoxia-inducible factor-1*α* (HIF-1*α*), IL-1*β*, fibronectin (FN), and collagen- (COL-) I proteins in the NRK-52E cells and target protein/*β*-ACTIN ratios, including HIF-1*α*/*β*-actin, IL-1*β*/*β*-actin, FN/*β*-actin, and COL-I/*β*-actin ratios (*n* = 4). Values are mean ± SE.

First, CCK8 assay was performed to identify the concentration that had no significant effect on NRK-52E activity for further experiments (Figure 4(a)). To confirm the effect of hypoxia on the expression of inflammatory factors in NRK-52E and the intervention effect of SSR, we subjected NRK-52E cells to hypoxia for 24 h. It was found that large quantities of IL-1*β* were secreted into the medium by NRK-52E cells under hypoxia, and a high expression of IL-1*β* was observed in the cytoplasm of NRK-52E cells (Figures 4(b), 4(c)). Western blotting showed that HIF-1*α*, IL-1*β*, FN, and COL-I were highly expressed in NRK-52E cells under hypoxia. However, treatment with SSR markedly decreased the secretion of IL-1*β* and suppressed the levels of HIF-1*α*, IL-1*β*, FN, and COL-I; the effect of SSR was dose dependent (Figure 4(d)). The results suggest that the expression and secretion of IL-1*β* in NRK-52E cells increased under the hypoxic environment, whereas SSR treatment had an ameliorative effect.

### 3.5. Shen Shuai II Recipe Inhibited the IL-1*β*/*c-Myc* Pathway in NRK-49F Cells

**Figure 5:**
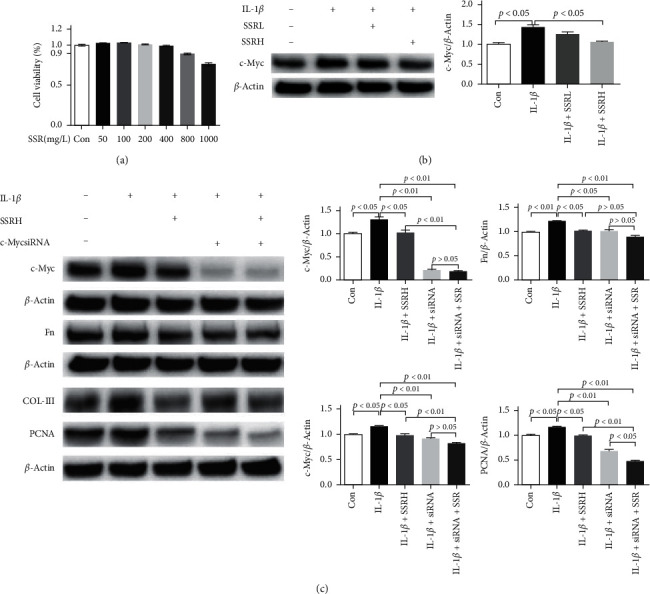
Shen Shuai II Recipe (SSR) inhibits the interleukin- (IL-) 1*β*/*c-Myc* pathway in NRK-49F cells. (a) Effect of SSR on the viability of NRK-49F cells (*n* = 5). (b) Representative western blotting band of *c-Myc* protein in NRK-49F cells and *c-Myc*/*β*-actin ratio (*n* = 4). (c) Representative western blotting band of *c-Myc*, fibronectin (FN), proliferating cell nuclear antigen (PCNA), and collagen- (COL-) III proteins in NRK-49F cells and target protein/*β*-actin ratios, including *c-Myc*/*β*-actin, FN/*β*-actin, COL-III/*β*-actin, and PCNA/*β*-actin ratios (*n* = 4). Values are mean ± SE.

To confirm that SSR has no antiproliferative and toxic effects on NRK-49F cells, we used the CCK8 assay to select the optimal SSR concentration (Figure 5(a)). In this study, *c-Myc* protein was highly expressed in the IL-1*β*-induced fibroblasts, and the SSRH group showed a significantly downregulated *c-Myc* protein expression (Figure 5(b)). To further investigate the role of the IL-1*β*/*c-Myc* pathway in NRK-49F cells, we used gene silencing of *c-Myc* by siRNA. Western blot results showed that *c-Myc* siRNA transfection successfully downregulated the expression of *c-Myc* in NRK-49F cells, and the inhibition of *c-Myc* protein expression was enhanced under the intervention of SSRH. Further observation showed that *c-Myc* knockdown reversed the increased protein expression of fibrosis-related factors FN, COL-III, and PCNA induced by IL-1*β* in NRK-49F cells. WB analysis also showed that combined treatment with SSR and gene silencing of *c-Myc* did not reduce the expression of FN and COL-I in NRK-49F as compared with *c-Myc* knockdown treated NRK-49F (Figure 5(c)).

## 4. Discussion

Although the pathological mechanism of renal fibrosis has been persistently investigated, to date, there is no ideal drug for the prevention and treatment of renal fibrosis. However, TCM shows an extensive application potential in the prevention and treatment of renal fibrosis. Specifically, SSR has been used in clinical practice for more than 20 years, and clinical studies have confirmed that SSR significantly improves the renal function and related complications in patients with CKD. However, the specific effective mechanism of SSR needs to be further explored. In the present study, we found that SSR significantly reversed the secretion of IL-1*β* and downregulated the expression of HIF-1*α*, *c-Myc*, and IL-1*β* proteins in residual renal tissue of rats with renal failure, which may be associated with the improvement of renal function and RIF.

Intrarenal hypoxia plays a key role in renal inflammatory reaction and renal interstitial fibrosis. HIF-1*α* is considered to be a key transcription factor in renal fibrosis induced by hypoxia. The close relationship between HIF-1*α* and renal interstitial inflammation may be related to the release of inflammatory cytokines from renal tubular cells or the cross talk between renal tubular cells and macrophages [21, 22]. Talwar et al. reported that the deletion of HIF-1*α* significantly reduced the lipopolysaccharide- (LPS-) induced production of proinflammatory factor IL-1*β* at the gene and protein level [23]. The 5/6 (A/I) rat model is a classic model of renal fibrosis; previous studies have confirmed that SSR improves renal hypoxia by increasing renal blood flow and improving oxygen consumption in 5/6 (A/I) rats [18, 19]. However, little is known about the mechanism by which SSR inhibits hypoxia-induced RIF. In the present study, we found that the expression of HIF-1*α* and IL-1*β* was significantly increased in NRK-52E cells cultured in hypoxic conditions and markedly inhibited by SSR treatment, suggesting that the effect of SSR is related to the release of inflammatory cytokines induced by HIF-1*α*. In addition, we also found that the expression of ECM proteins such as FN and COL-I increased in NRK-52E cells, which may be related to epithelial-mesenchymal transdifferentiation [24]; however, the specific mechanism remains to be further investigated.

Inflammation is a complex reaction that occurs when the body is subjected to harmful stimulation. During organ fibrosis, a large number of inflammatory cells infiltrate the tissue, and cytokines and chemokines interact with each other. It causes apoptosis and necrosis of cells in the organ, activation of fibroblasts, and production of large quantities of ECM, resulting in increased fibrous tissue and organ dysfunction. Inflammation-induced renal fibrosis is an important factor in the progression of CKD [25]. Previous clinical studies have shown that chronic inflammation has a strong correlation with long-term mortality and morbidity in patients with end-stage renal disease [26, 27]. IL-1*β* is recognized as one of the inflammatory factors closely associated with renal interstitial fibrosis. Immune cells or nonimmune cells (such as renal tubular epithelial cells) secreted mature into body fluids after recognizing pathogens or molecular patterns of injury; then, through recruitment, IL-1*β* binds to IL-1R at the site of injury to amplify the inflammation [25]. Recent studies have shown that IL-1*β* induces myofibroblast differentiation and ECM deposition through IL-1R and IRAK4 signal transduction complexes, which is the catalyst for the formation and progression of RIF [28]. Previous in vivo experiments suggest that SSR significantly inhibits the NLRP3/Asc/caspase-1/IL-1*β* cascade reaction and secretion of IL-1*β* in the 5/6 (A/I) rat model [29]. On the basis of previous research, this study demonstrated that hypoxia increases the expression and secretion of IL-1*β* in NRK-52E cells. As we expected, the expression of IL-1*β* in vivo and in vitro was decreased in a dose-dependent manner under SSR treatment. Further experiments confirmed the direct effect of IL-1*β* on the proliferation of fibroblasts, which could be reversed by gene silencing of *c-Myc*.

*c-Myc* is a multifunctional transcription factor and is considered to be the inducer and accelerator of cell metabolism and proliferation [30, 31]. The relationship between *c-Myc* and renal fibrosis has recently attracted increasing attention. *c-Myc* protein expression has been observed to be significantly upregulated in various animal models of renal fibrosis [9, 10]. A recent study reported that the use of a *c-Myc* inhibitor, 10058-F4, significantly attenuated renal fibrosis in vivo, and in vitro experiments confirmed that *c-Myc* inhibitors and siRNA silencing of *c-Myc* blocked IL-1*β*-induced fibroblast proliferation and activation [10]. Lemos et al. found that *c-Myc*-mediated glycolysis plays a central role in energy metabolism in patients with chronic renal failure, which confirms the key role of the IL-1*β*/*c-Myc* pathway in this process and clarifies the relationship between inflammation, glycolysis, and RIF [11]. Consistent with previous findings, the results of the present study showed that *c-Myc* protein expression increased in the 5/6 (A/I) rat model and IL-1*β*-induced fibroblasts. Gene silencing of *c-Myc* reversed the increased expression of PCNA and the ECM proteins and also blocked or attenuated the antifibrotic effect of SSR in IL-1*β*-induced NRK-49F cell, suggesting that *c-Myc* is involved in the progression of RIF and is an important target for SSR in the improvement of renal fibrosis. These results indicate that SSR improves the proliferation and activation of renal fibroblasts through the IL-1*β*/*c-Myc* pathway. However, further research is needed to identify the factors downstream of *c-Myc* that promote renal fibrosis.

## 5. Conclusions

We confirmed that SSR ameliorates the effects of hypoxia in NRK-52E cells and inhibits the development of RIF by inhibiting IL-1*β*/*c-Myc*-dependent proliferation and excessive production of ECM in NRK-49F cells. The findings suggest that SSR alleviates RIF by improving hypoxia via the IL-1*β*/*c-Myc* pathway. Therefore, SSR may be a potential drug for the treatment of RIF in patients with chronic renal failure.

## Figures and Tables

**Table 1:** Composition of Shen Shuai II Recipe.

Herb	English name	Medicinal part	Proportion (g)
*Codonopsis pilosula* (Franch.) Nannf. (Dangshen)	Codonopsis Radix	Rhizome	15
*Epimedium brevicornum* Maxim. (Yinyanghuo)	Epimedii Folium	Leaf	15
*Salvia miltiorrhiza* Bge. (Danshen)	Salviae Miltiorrhizae Radix et Rhizoma	Root and rhizome	15
*Angelica sinensis* (Oliv.) Diels (Danggui)	Angelicae Sinensis Radix	Root	15
*Rheum palmatum* L. (Dahuang)	Rhei Radix et Rhizoma	Root and rhizome	15
*Perilla frutescens* (L.) Britt. (Zisuye)	Perillae Folium	Leaf	15
*Coptis chinensis* Franch. (Huanglian)	Coptidis Rhizoma	Rhizome	6
*Ligusticum chuanxiong* Hort. (Chuanxiong)	Chuanxiong Rhizoma	Rhizome	15
*Prunus persica* (L.) Batsch (Taoren)	Persicae Semen	Seed	15

**Table 2:** Related information of compounds in Shen Shuai II Recipe.

Compound	Formula	Molecular weight	M + H/M − Cl+	Rt (min)	M − H	Rt (min)
Icariin	C_33_H_40_O_15_	676.23617	677.24399	6.54	675.22834	—
Tanshinone IIA	C_19_H_18_O_3_	294.13	295.13287	12.37	293.11722	—
Berberine hydrochloride	C_20_H_18_ClNO_4_	371.09188	336.12303	6.43	370.08406	—
Ferulic acid	C_10_H_10_O_4_	194.05736	195.06518	5.25	193.04953	—
Amygdalin	C_20_H_27_NO_11_	457.16	458.16568	—	502.1568	4.03
Salvianolic acid B	C_36_H_30_O_16_	718.15	719.16066	—	717.14501	5.80
Emodin	C_15_H_10_O_5_	270.05227	271.06009	—	269.04444	9.96
Codonopsis lactone	C_15_H_20_O_3_	248.14070	249.14852	9.40	247.13287	—
Caffeic acid	C_9_H_8_O_4_	180.04171	181.04953	—	179.03388	4.25
Rosmarinic acid	C_18_H_16_O_8_	360.08396	361.09179	—	359.07614	5.64

## Data Availability

The datasets used and/or analyzed during the present study are available from the corresponding author upon reasonable request.
